# Game, Set… Revision! A Case Report of a Tennis Player Who Smashed His Scaphotrapeziotrapezoid-Joint Pyrocardan Implant Twice

**DOI:** 10.1016/j.jhsg.2026.100964

**Published:** 2026-02-23

**Authors:** Christos Ni. Sarigiannis, Peter Axelsson

**Affiliations:** ∗ Department of Hand Surgery, Sahlgrenska University Hospital, Gothenburg, Sweden; † Institute of Clinical Sciences, Sahlgrenska Academy, University of Gothenburg, Gothenburg, Sweden

**Keywords:** Arthroplasty, Implant failure, Pyrocardan, revision, STT joint

## Abstract

A healthy middle-aged man was treated for isolated scaphotrapeziotrapezoid joint osteoarthritis with implant arthroplasty using a Pyrocardan implant. We report implant breakage twice during tennis training, and the debut of squeaking after the first failure. The implant was revised and replaced both times. Revision of the implant resolved the squeaking. To our knowledge, this is the first report of a type of complication with this implant, indicating potential limitations for its future use.

Osteoarthritis (OA) of the scaphotrapeziotrapezoid (STT) joint is a relatively common finding in cadaveric specimens.^1^ However, in clinical practice, it is an uncommon isolated cause of wrist pain, and the need for surgical intervention in symptomatic isolated STT OA is rare. Surgical treatment options for isolated STT arthritis include excision of the trapezium with or without ligament reconstruction and tendon interposition, fusion of the STT joint, arthroscopic debridement, excision of the distal scaphoid, and pyrocarbon implant arthroplasty.^2^, ^3^, ^4^

Among all treatment alternatives, implant arthroplasty is the one that preserves best wrist kinematics by maintaining scaphoid length.^4^ However, reported outcomes are based on small cohorts with variable results.^3^^,^^5^ The availability of pyrocarbon implants for this indication is limited, and their limitations are yet to be fully explored. Specifically, for the Pyrocardan implant (Stryker, Kalamazoo, Michigan, USA), this represents an off-label indication, as the implant originally was designed for the first carpometacarpal joint. Nevertheless, its application in the STT joint has been described previously in the literature.^4^^,^^5^ Therefore, this case report of a complication is important for evaluating future indications of the Pyrocardan implant in STT OA.

Written informed consent was obtained from the patient for publication of this case report and accompanying images and video. The case report cohered to the CARE guidelines.

## Case Report

### Patient background

A 48-year-old, healthy, physically active man employed in administrative work was referred to our department with a two-year history of radial-sided wrist pain in his dominant hand. The pain was provoked primarily by activity but later also present at rest. Because of symptom progression, he had discontinued participation in recreational tennis. There was no history of trauma. The patient previously had undergone physiotherapy, which yielded no clinical improvement.

### Preoperative assessment

On clinical examination, tenderness was elicited over the STT joint, exacerbated by radial deviation. Hand grip strength measured 52 kg on the dominant side and 54 kg on the nondominant side. No difference in the range of motion was observed between the two wrists. A magnetic resonance imaging scan obtained before referral demonstrated isolated STT joint arthritis, without involvement of adjacent carpal joints or evidence of ligamentous injury. Additionally, an interosseous ganglion within the scaphoid was identified. The patient received a corticosteroid injection into the STT joint under fluoroscopic guidance, and a computed tomography (CT) scan subsequently was ordered for further evaluation.

On a follow-up visit 2 months later, he demonstrated a good initial response to the injection, with partial regression of symptoms. The CT scan confirmed isolated STT arthritis and revealed that the scaphoid cyst was larger than previously appreciated, with subchondral extension to the STT joint. Given these findings, a two-stage surgical approach was planned. The first stage would involve curettage of the cyst and bone grafting. Following adequate healing, the second stage would include STT joint resurfacing with a Pyrocardan implant.

### First-stage surgical treatment: bone grafting

**Figure 1:**
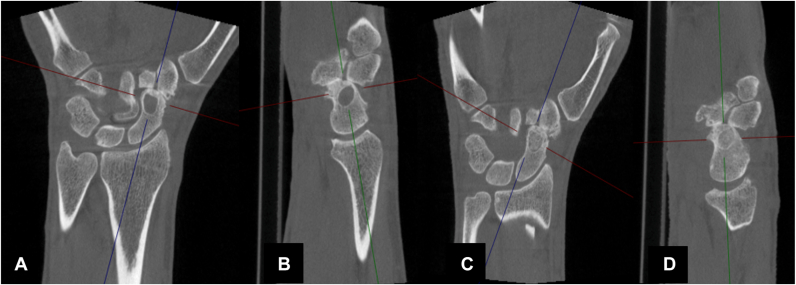
**A, B** Preoperative CT scan. **C, D** CT scan 3 months after bone packing. CT, computed tomography.

A volar open approach to the scaphoid was performed, followed by curettage of the bone cyst and packing of the defect using a cancellous bone graft harvested from the distal radius. After surgery, the wrist was immobilized in a cast for 5 weeks, followed by the use of a prefabricated orthosis during risk activities. Radiologic follow-up at 3 months confirmed complete healing of the bone defect. Figure 1 shows the preoperative CT scan and the scan obtained 3 months after bone grafting.

### Second-stage surgical treatment: pyrocarbon implant arthroplasty

**Figure 2:**
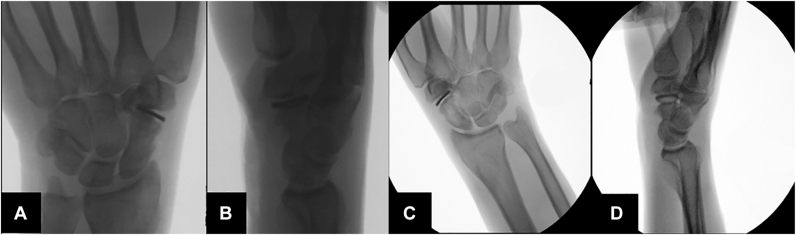
Perioperative fluoroscopic x-rays. **A, B** First implantation. **C, D** Second revision.

Ten months after the initial procedure, the patient proceeded with the second stage of surgical treatment. The same volar incision was used. The proximal portion of the trapezoid was excised. A size 15 Pyrocardan implant was implanted, with the M1 mark oriented toward the trapezium and the TR mark toward the scaphoid. After surgery, the patient was immobilized in a cast for 3 weeks. Figures 2A,B show the perioperative fluoroscopic x-rays.

### Failure of the initial pyrocarbon implant

**Figure 3:**
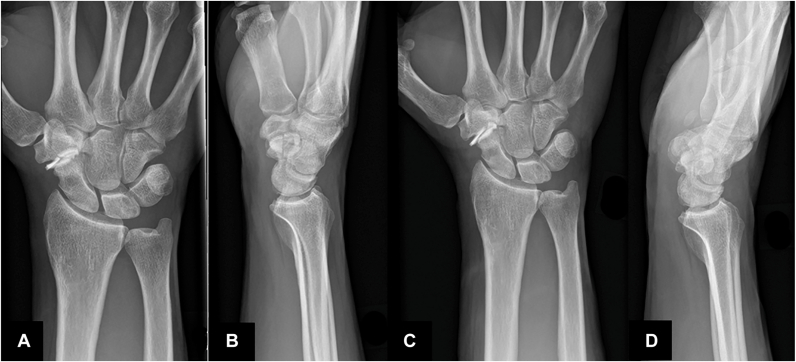
X-rays of implant failure. **A, B** First failure. **C, D** Second failure.

The postoperative outcome was favorable, and the patient remained completely symptom-free for approximately 14 months. He was able to resume all activities he had discontinued previously because of pain. However, during a tennis match at recreational level, he experienced a sudden onset of pain at the base of the thumb while serving. He reported a distinct sensation of mechanical failure followed by debut of ‘squeaking’. A video is available in the supplementary material (Video 1, available online on the *Journal*’s website at https://www.jhsgo.org). Radiographic imaging confirmed implant breakage (Fig. 3).

**Figure 4:**
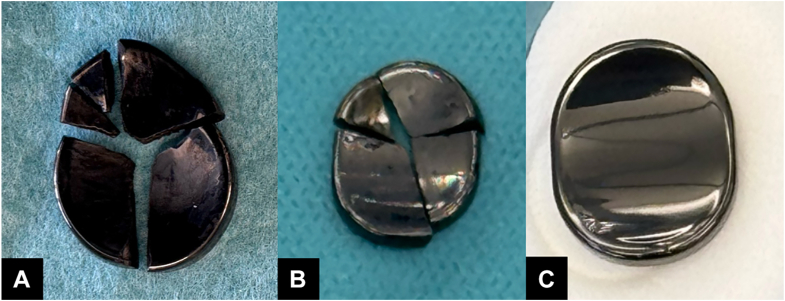
Perioperative photographs of the failed implants at the **A** first and **B** second revisions. **C** A photograph of the new implant before implantation is shown for comparison.

Revision surgery was performed through the same volar approach. Pyrocarbon debris was debrided and washed out. As the joint surfaces looked smooth with no peak and the implant had performed well until breakage no further resection was performed. The broken implant was replaced with a new Pyrocardan implant of the same size. An intraoperative photograph of the fractured implant is shown in Figure 4. The same postoperative immobilization protocol was followed.

### Failure of the revised pyrocarbon implant

The postoperative course initially was uneventful, and the patient remained symptom-free for 8 months. The squeaking decreased notably at the six-week follow-up and had resolved by 12 weeks. He was instructed to avoid excessive loading of the wrist and refrained from competitive tennis. However, he engaged in light tennis training under controlled conditions, with supervision from a coach and wrist taping. Following a gentle forehand stroke, he experienced the same acute onset of pain and mechanical failure as reported previously. No difference in the squeaking was noted this time. Radiographs again confirmed implant failure (Fig. 3).

A second revision surgery was performed through the previous incision. Visible pyrocarbon debris was debrided, and no further bone resection was performed. The implant was exchanged to one of the same size. The failed implant is shown in Figure 4.

### Deep infection after second implant revision

Unfortunately, within 2 weeks after surgery, the patient experienced a deep surgical site infection caused by *Staphylococcus* *epidermidis*, necessitating removal of the implant. Initial treatment included intravenous antibiotics, followed by oral antibiotics for a total duration of 4 weeks. After implant removal, the wrist was immobilized with a cast for 2 weeks. Upon cast removal, the patient was instructed to avoid heavy lifting during the early recovery phase and to use a wrist brace. At the most recent follow-up, 2 months after implant removal, the patient was pain-free and exhibited no signs of active infection. Fluoroscopic evaluation demonstrated preserved joint space in the STT joint without evidence of collapse during movement. The patient was advised to use the hand as tolerated and is scheduled for reevaluation in 2 months to discuss the potential need for prosthesis reimplantation or alternative salvage procedures.

## Discussion

Patients with isolated, symptomatic STT OA are relatively uncommon in clinical practice, and evidence guiding the optimal surgical treatment approach remains limited. Achieving stability and mobility of the STT joint is essential for patient satisfaction and functional outcomes.^4^ In a recent review, Bellemère et al.^4^ recommended implant arthroplasty using the Pyrocardan implant as the treatment of choice. A case series involving 13 patients with a minimum follow-up of 5 years reported no implant failures and remains the most comprehensive clinical evaluation of this implant to date.^5^ However, given the limited availability of suitable implants, reporting complications and limitations associated with Pyrocardan use is crucial to guide future clinical decision-making.

The Pyrocardan implant features a biconcave design with a central thickness of 1 mm, regardless of implant size.^4^ In our case report, both failed implants exhibited fracture lines through the center of the prosthesis, suggesting that central loading may have contributed to structural failure. A 3-dimensional biomechanical model^6^ of wrist load transmission has shown that more than half of the axial load can be directed through the STT joint, particularly in wrist extension, which shifts loading radially. Activities, such as serving and forehand strokes in tennis, further concentrate force through the STT joint,^7^^,^^8^ potentially increasing stress on implants placed in this location causing the failure. Based on our findings, we suggest that the Pyrocardan implant may be contraindicated in patients with symptomatic STT OA who intend to continue high-load activities, particularly those involving wrist extension under load, such as tennis.

Another notable observation was the onset of squeaking following implant breakage. Squeaking is a known minor complication of pyrocarbon implants used in hand surgery.^9^ It usually is benign and resolves spontaneously.^9^ We identified only one case report of debut of squeaking due to implant failure in an metacarpophalangeal joint.^10^ To our knowledge, this is the first report of squeaking for an implant in the STT joint, and the first report of debut of squeaking due to implant failure.

A final consideration is whether pyrocarbon debris increases the risk of infection. In both revision surgeries, all visible debris was debrided thoroughly. A brief literature search did not identify any evidence suggesting an increased infection risk directly attributable to pyrocarbon debris, though published data on this specific issue remain limited.

## Declaration of Generative AI and AI-assisted technologies in the writing process

During the preparation of this work, the authors used ChatGPT-EDU (OpenAI) only for language editing in order to improve the readability of the text. After using this tool, the authors reviewed and edited the content and take full responsibility for the content of the publication.

## Conflicts of Interest

No benefits in any form have been received or will be received related directly to this article.

## Supplementary Data

Video 1 Audible squeaking after implant failure.
